# Liver transplantation can prevent the progression of neurological damage in hyperornithinemia-hyperammonemia-homocitrullinuria syndrome and maintain long-term metabolic stability — The largest single-center experience

**DOI:** 10.1186/s13023-025-04077-5

**Published:** 2025-10-22

**Authors:** Hongfei Ju, Jun Wang, Xinyue Wang, Wei Qu, Zhigui Zeng, Ying Liu, Haiming Zhang, Li-Ying Sun, Lin Wei, Zhi-Jun Zhu

**Affiliations:** 1 Liver Transplantation Center, National Clinical Research Center for Digestive Diseases, Beijing Friendship Hospital, Capital Medical University, Beijing, China; 2 Department of Critical Liver Disease, National Clinical Research Center for Digestive Diseases, Beijing Friendship Hospital, Capital Medical University, Beijing, China; 3 Clinical Center for Pediatric Liver Transplantation, Capital Medical University, Beijing, China; 4 State Key Lab of Digestive Health, Beijing, China; 5 Laboratory for Clinical Medicine, Capital Medical University, Beijing, China; 6 Beijing Key Laboratory of Tolerance Induction and Organ Protection in Transplantation, Beijing, China; 7 Intensive Care Unit, Beijing Tongren Hospital, Capital Medical University, Beijing, China; 8 Liver Transplantation Center, National Clinical Research Center for Digestive Diseases, State Key Lab of Digestive Health, National Clinical Research Center for Digestive Diseases, Beijing Friendship Hospital, Capital Medical University, Beijing, China

**Keywords:** Hyperornithinemia-hyperammonemia-homocitrullinuria (HHH) syndrome, Liver transplantation, Urea cycle disorder

## Abstract

**Background:**

Hyperornithinemia-Hyperammonemia-Homocitrullinuria (HHH) syndrome is a rare urea cycle disorder caused by mutations in the SLC25A15 gene, leading to metabolic and neurological impairments. Liver transplantation (LT) may restore urea cycle function and prevent disease progression.

**Methods:**

This retrospective study analyzed six patients with HHH syndrome who underwent LT between 2016 and 2022. Pre- and post-transplant evaluations included biochemical tests, genetic analysis, neurological assessments, and quality-of-life measures.

**Results:**

LT successfully normalized metabolic parameters (ammonia and amino acid levels) and allowed patients to resume normal diets. Early transplantation resulted in neurological improvement in 5 of 6 patients (83.3%), including reduced lower limb spasticity and improved walking ability. Two patients (33.3%) achieved nearly normal gait, and one patient (16.7%) recovered to normal motor function within three months after LT. Quality-of-life scores improved in 2 patients (33.3%). The overall survival rate was 83.3%, with one patient dying from unrelated causes 5 years post-transplant. No significant long-term complications were observed in the surviving patients.

**Conclusions:**

Liver transplantation is an effective treatment for HHH syndrome, halting neurological decline and improving quality of life. Early LT before irreversible damage provides the best outcomes, making it a viable option for patients with progressive symptoms unresponsive to conventional therapies.

**Clinical trial number:**

Not applicable.

**Supplementary Information:**

The online version contains supplementary material available at 10.1186/s13023-025-04077-5.

## Introduction

**Fig. 1:**
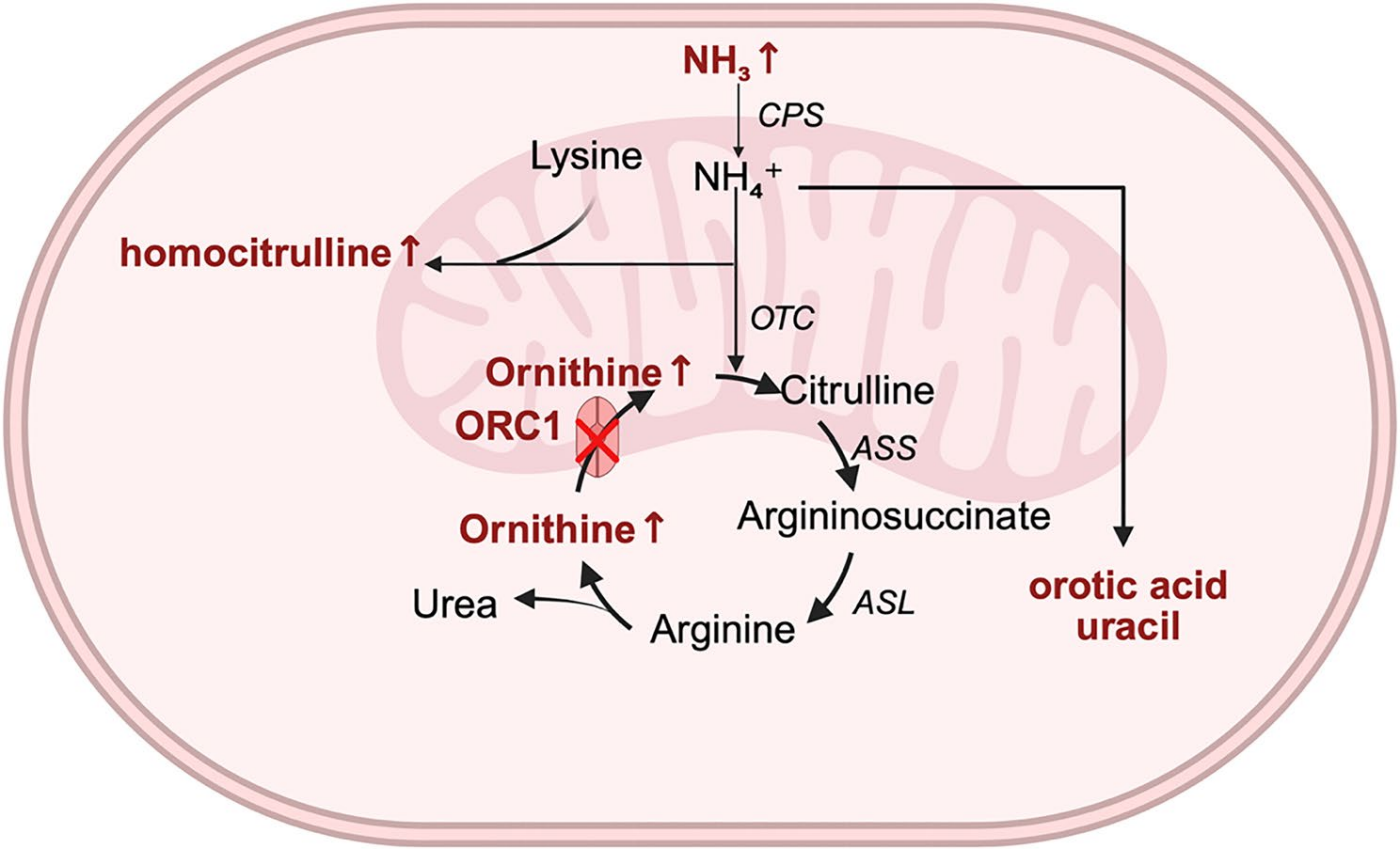
The urea cycle and related pathways. The urea cycle occurs partially in the mitochondria and partially in the cytosol. First, within the mitochondria, ammonia combines with carbon dioxide to form carbamoyl phosphate. Under the action of ornithine transcarbamylase, carbamoyl phosphate then condenses with ornithine to form citrulline, which is transported through the mitochondrial membrane into the cytosol. In the cytosol, citrulline combines with aspartate under the action of argininosuccinate synthetase to produce argininosuccinate, which is then cleaved by argininosuccinate lyase to form arginine. Arginine is subsequently broken down by arginase to regenerate ornithine and produce non-toxic urea. The cytosolic ornithine is then transported back into the mitochondria via ORNT1 to initiate another cycle. When ORNT1 function is impaired, the following biochemical changes occur: (1) mitochondrial ornithine levels decrease, while blood ornithine levels increase; (2) ornithine cannot react adequately with carbamoyl phosphate, leading to carbamoyl phosphate accumulation; (3) accumulated carbamoyl phosphate is diverted to alternative pathways, producing orotic acid, uracil and forming homocitrulline with lysine, resulting in elevated homocitrulline; (4) due to disruption of the urea cycle, free ammonia accumulates, leading to hyperammonemia and associated clinical symptoms. The abbreviations correspond to: CPS, carbamyl-phosphate synthetase; OTC ornithine transcarbamylase; ORC1, ornithine carrier 1; ASS argininosuccinate synthetase; ASL argininosuccinate lyase

Hyperornithinemia-hyperammonemia-homocitrullinuria (HHH, MIM#238970) syndrome is a rare autosomal recessive disorder of the urea cycle caused by mutations in the SLC25A15 gene (MIM*603861), which encodes for the mitochondrial ornithine carrier (ORC1) [1]. The function of this carrier is complex and varies according to the specific tissue in which it operates. In hepatocytes, ORC1 is responsible for an important transport step in the urea cycle by catalyzing the electroneutral exchange of cytoplasmic ornithine for matrix citrulline, which links urea cycle enzyme activities in the cytoplasm and matrix [2]. When the function of the ORC1 is impaired, mitochondrial ornithine levels decrease while blood ornithine levels increase. Consequently, ornithine may be diverted into alternative metabolic pathways, leading to the formation of pyruvic acid and homocitrulline, which causes homocitrullinuria. This block in the urea cycle results in the accumulation of free ammonia, leading to hyperammonemia (Fig. 1) [3].

The clinical manifestations of this syndrome can vary widely among patients. Common symptoms include aversion to protein-rich foods, recurrent vomiting, liver enlargement, abnormal liver function, and neurological impairments such as drowsiness, intellectual disability, learning difficulties, epilepsy, ataxia, and spastic paraplegia [4]. In acute cases, symptoms may escalate to include severe vomiting, acute liver failure, altered consciousness, and coma [5].

Long-term management typically involves a low-protein diet, supplementation with arginine or citrulline, and the use of ammonia scavengers like sodium phenylacetate or sodium benzoate [1]. Patients receiving conservative treatment generally achieve metabolic stability and do not experience recurrent hyperammonemia. However, the progression of spastic paraplegia may continue despite these interventions [6]. For severe liver-based metabolic disorders, such as urea cycle disorders (UCDs), liver transplantation or hepatocyte transplantation can effectively restore enzyme function [7]. To date, liver transplantation has been reported in only two cases of severe metabolic disorder associated with HHH syndrome [8, 9].

**Fig. 2:**
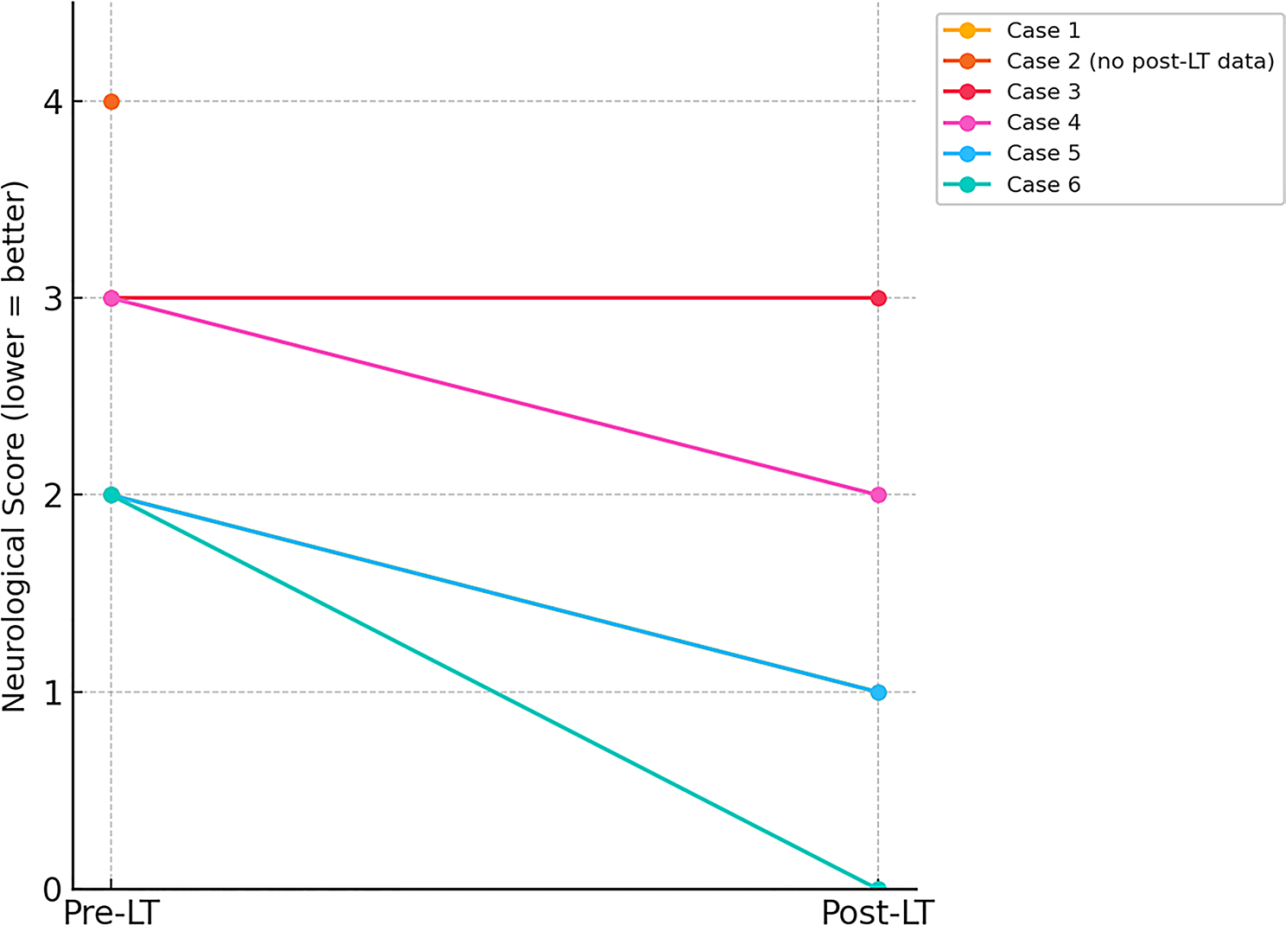
Changes in neurological function scores before and after liver transplantation (LT) in six patients with hyperornithinemia-hyperammonemia-homocitrullinuria syndrome. Each line represents one patient’s score change from pre-LT to post-LT; a lower score indicates better neurological function. Case 2 lacked postoperative neurological assessment due to death from unrelated causes and is shown with only a pre-LT data point

In this report, we present the characteristics and clinical outcomes of six patients with HHH syndrome who underwent LT at our center. We include detailed descriptions of individualized treatment plans and clinical presentations, which may provide valuable insights into the management of this complex condition. This information not only enhances our understanding of the underlying pathophysiological mechanisms but also supports timely and effective interventions for affected patients (Fig. 2).

## Methods

### Study population

We conducted a retrospective analysis of six patients with HHH syndrome, comprising five males and one female. These patients were admitted to the Liver Transplantation Center at Beijing Friendship Hospital of Capital Medical University between January 2016 and September 2022. Blood ornithine, citrulline and arginine levels were measured using liquid chromatography tandem mass spectrometry, and genetic testing was performed on all patients to confirm mutations in the SLC25A15 gene. All organs used in this study were obtained from voluntary donors after informed consent or appropriate authorization, in full compliance with national regulations and ethical guidelines. All liver transplantation procedures received approval from the Ethics Committee of Beijing Friendship Hospital.

The indications for LT included progressive spastic paraparesis and acute liver failure, despite conventional treatment. Patient evaluations were conducted using the transplantation scoring system described by Kasahara et al. [10]. Data on preoperative and postoperative blood ammonia levels, blood ornithine concentrations, liver function, and clinical manifestations were collected to assess the outcomes of the transplantation. Additionally, we utilized a grading scale based on the work of Morioka et al. to evaluate neurological status, physical growth, and quality of life for patients during follow-up visits at the clinic [11].

The study was conducted in accordance with the Declaration of Helsinki (as revised in 2013). This study was approved by the Ethics Committee of Beijing Friendship Hospital, Capital Medical University (No. 2020-P2-094-01). Informed consents were obtained from all legal guardians of participants included in the study.

### Statistical analysis

Data are presented as mean ± standard deviation (SD). The small number of participants in this study precluded use of formal statistical analyses.

## Results

### Preoperative clinical characteristics and managements

Genetic analysis revealed homozygous pathogenic variants in the SLC25A15 gene in four of the patients (cases 1, 3, 5, and 6). Specifically, case 1 exhibited a missense mutation, c.416 A > G (p.E139G), with his father being heterozygous and his mother displaying the wild-type genotype. Chromosomal microarray analysis was performed on the proband and their parents, which identified a 90 MB region of segmental uniparental isodisomy on chromosome 13 (13q12.11–q34; chr13:21,735,988–112,715,600), thereby confirming the diagnosis of HHH syndrome. The remaining two patients (cases 2 and 4) presented with compound heterozygous mutations (Table 1).

**Table 1:** Patient demographics and genetic mutation information

Case	Gender	Age at presentation (months)	Age at LT (months)	Gene mutations	Zygosity	Mutation at protein level	Transplantation score
1	Male	20	72	c.416 A > G + paternal uniparental disomy of 13q12.11q34	segmental paternal uniparental isodisomy of 13q12.11–q34	p.E139G	18
2	Male	16	21	c.197G > T/c.535 C > T	Compound heterozygous	p.G66V/p.R179*	25
3	Male	8	104	c.535 C > T	Homozygous	p.G66V	15
4	Male	15	43	c.165 C > A/c.535 C > T	Compound heterozygous	p.Y55*/p.R179*	17
5	Female	26	29	c.535 C > T	Homozygous	p.G66V	15
6	Male	6	28	c.823 C > T	Homozygous	p.R275*	17

“*” indicates a nonsense mutation

LT liver transplantation

Case 1 had a 90 Mb paternal uniparental disomy of chromosome 13q12.11q34 detected by chromosomal microarray analysis

All six patients displayed significant developmental delays in motor skills, being unable to lift their heads, sit, roll over, or crawl according to typical developmental milestones. Apart from cases 3 and 6, likely due to their younger ages, the other four patients exhibited aversion to protein-rich foods, and case 2 also experienced recurrent vomiting. All patients had spastic paraparesis, with case 2 additionally suffering from myoclonic seizures affecting the left upper limb. Three patients (cases 1, 4, and 5) demonstrated a “tip-toe” gait and could only walk short distances, while cases 2 and 6 were completely unable to ambulate. Among the four children older than 12 months, varying degrees of dysarthria and delayed speech were observed. All five patients, except case 2, exhibited chronic liver function abnormalities characterized by elevated transaminases, and three of them showed mild coagulation abnormalities.(Table 2). For case 2, complete preoperative blood amino acid and urinary organic acid profiles were not available due to the loss of the original laboratory report; however, medical records confirmed that the preoperative plasma ornithine level was markedly elevated above the reference range. Liver ultrasonography revealed increased echogenicity and varying degrees of hepatic enlargement in these children prior to liver transplantation, at ages ranging from 21 to 104 months. Notably, case 2 presented with fulminant hepatic failure, requiring aggressive supportive therapy.

**Table 2:** Comparison of biochemical parameters Pre- and Post-Liver transplantation

Result	Case1		Case2^*^		Case3		Case4		Case5		Case6		RV
	Pre-LT	Post-LT	Pre-LT	Post-LT	Pre-LT	Post-LT	Pre-LT	Post-LT	Pre-LT	Post-LT	pre-LT	Post-LT	
Ammonia (µmol/L)	108	24	206	40	46	25	70	44	84	31	48	34	< 40
Ornithine (µmol/L)	367.4	30.21	↑		262.8	54.46	184.09	102.89	135.2	51.95	158.76	101.69	30–110
Citrulline (µmol/L)	28.9	3.78			30.5	4.68	10.15	9.72	14.17	4.77	29.07	14.06	10–52
Arginine (µmol/L)	35.8	6.39			11	2.43	2.27	2.7	4.33	2.39	12.27	1.69	20–112
Orotic acid (mmol/mol Cr)	29.75	0			NA	0	466.37	0	0	1	55.22	0	0.00-1.50
Uracil (mmol/mol Cr)	63.64	5.47			NA	0	187.91	9.94	31.6	8.95	107.44	1.6	0.00–7.00
INR	1.07		2.36		1.19		1.32		1.32		1.22		0.8–1.2
Prealbumin(mg/L)	174		130.7		152.4		177		137		122		170–420

^*^ Case 2 underwent relevant tests before the operation, but the report was lost. According to the medical records, the preoperative ornithine level in the blood exceeded the reference range

LT liver transplantation, RV reference value

Before LT, all patients received conservative management, which included a low-protein diet supplemented with arginine. Cases 2 and 5 also received citrulline. Additionally, cases 5 and 6 were treated with ammonia scavengers: sodium benzoate for case 5 and sodium phenylbutyrate for case 6 (Table 3). Despite these interventions, metabolic abnormalities were not adequately corrected. Prior to transplantation, we conducted an analysis of blood amino acids and urinary organic acids in all patients using tandem mass spectrometry (Table 2). This analysis revealed elevated plasma ornithine levels, as well as increased urinary uracil and orotate in all patients. Neurological impairment continued to progress in all cases, including worsening spastic paralysis of the lower limbs (Table 3).

**Table 3:** Patient clinical manifestations and treatment plans

Case	Manifestation	Conservative treatments	Procedure
1	spastic paralysis, intellectual disability, dysphasia, movement and gait disturbances, aversion for protein rich foods, increase of trans aminases	low-protein diet, supplemented with arginine	DDLT
2	fulminant liver failure, spastic paralysis, myoclonic seizures, movement and gait disturbances, aversion for protein rich foods, recurrent vomiting	low-protein diet, supplemented with citrulline and arginine, sodium phenylbutyrate	LDLT
3	spastic paralysis, intellectual disability, dysphasia, movement and gait disturbances, increase of trans aminases	low-protein diet, supplemented with arginine	DDLT
4	spastic paralysis, dysphasia, movement and gait disturbances, aversion for protein rich foods, increase of trans aminases	low-protein diet, supplemented with arginine	LDLT
5	spastic paralysis, intellectual disability, movement and gait disturbances, aversion for protein rich foods, increase of trans aminases, coagulation abnormalities	low-protein diet, supplemented with citrulline and arginine, sodium phenylbutyrate	DDLT
6	spastic paralysis, developmental delay, increase of trans aminases	low-protein diet, supplemented with arginine	DDLT

DDLT deceased donor liver transplantation, LDLT living donor liver transplantation

### Liver transplantation and postoperative outcomes

All patients underwent LT due to progressive neurological injury despite conventional treatment, with each presenting a transplantation score exceeding 10, indicating a clear indication for the procedure. Among the six patients, two received living donor liver transplantation (LDLT), while the remaining four underwent deceased donor liver transplantation (DDLT). All procedures were performed using standard surgical techniques (Table 3). The mean age at the time of transplantation was 49.5 ± 32.3 months. In the LDLT group, the donors were the patients’ heterozygous parents. Initial immunosuppressive therapy included tacrolimus, mycophenolate mofetil, and low-dose methylprednisolone, with the latter gradually tapered over three months and the former over one year.

Postoperatively, all patients transitioned to a normal diet without protein restrictions within three to seven days after surgery. Blood ammonia levels remained stable and within the normal range for an extended period following transplantation. Plasma amino acids and urinary organic acid levels were reassessed 14 to 28 days after surgery, revealing that metabolic abnormalities, including elevated plasma ornithine and urinary uracil and pyruvic acid levels in some patients, had returned to the reference range (Table 2).

Postoperative complications included cytomegalovirus infection in case 1, bile leakage requiring secondary surgical intervention in case 4, and bile leakage in case 6, which improved with drainage via percutaneous catheter placement. No complications classified as Clavien-Dindo I or higher were observed in the other patients. The follow-up period for patients ranged from 22 to 101 months. Case 2 passed away five years post-LT due to unrelated causes, while the remaining patients are alive. The overall survival rate for both patients and grafts is currently 83.33% (Table 4).

**Table 4:** Complications post- liver transplantation and outcomes

Case	Complications	Procedure	Clavien-Dindo Classification	Outcomes
1	Cytomegalovirus infection	Drug	II	Survive
2	None	None	None	Death
3	None	None	None	Survive
4	Bile leak	Surgery	Ⅲb	Survive
5	None	None	None	Survive
6	Bile leak	percutaneous drainage	Ⅲa	Survive

After LT, all patients demonstrated varying degrees of improvement in neurological function (Table 5). Case 6 is now able to walk almost normally, while patients 1, 3, 4, and 5 have experienced significantly reduced muscle tone in their lower limbs compared to preoperative levels, leading to improvements in gait. Although patient 3 still exhibits a “tip-toe” gait, he can now walk longer distances without assistance.

**Table 5:** Neurological function and quality of life pre-/post- liver transplantation

Case	Neurological status, pre-/post-LT	Quality of life, pre-/post-LT
1	2/1	Fair/Fair
2	4/-	Poor/-
3	3/3	Poor/Poor
4	3/2	Poor/Fair
5	2/1	Fair/Fair
6	2/0	Fair/Good

LT liver transplantation

All patients became capable of effective communication, with some able to speak in simple sentences. Those with severe intellectual disabilities can now respond to simple verbal requests from their parents and express excitement in the presence of family or familiar friends. Case 1 attends school and demonstrates age-appropriate social skills and psychomotor abilities, with no apparent learning disabilities. Case 6 has fully recovered but has not yet reached school age.

During the follow-up assessment, two patients showed significant improvements in their quality of life, while progress in the remaining patient was primarily linked to enhanced motor function, as reflected in their quality of life assessment scores (Table 5).

## Discussion

UCDs are rare inherited metabolic disorders, and HHH syndrome accounts for only 1-3.8% of the reported UCDs [12, 13]. The acute manifestations of HHH syndrome include encephalopathy, altered levels of consciousness, and seizures, with some patients experiencing fulminant hepatic failure and coagulation abnormalities. Chronic symptoms often involve aversion to protein-rich foods, recurrent vomiting, intellectual disability, and developmental delays [14]. A notable characteristic of HHH syndrome is the progressive worsening of neurological function, primarily manifested as spastic paraparesis and signs of cone-bundle injury [15].

Long-term management typically involves a low-protein diet supplemented with arginine or citrulline, potentially combined with ammonia scavengers like sodium benzoate or sodium phenylbutyrate. The long-term prognosis can vary significantly, ranging from mild neurological impairment to severe disability. Furthermore, many patients do not respond well to conservative treatment, leading to severe neurological disabilities and episodes of metabolic decompensation due to strict dietary controls. Current evidence does not establish a clear correlation between neurological outcomes and factors such as age at onset, genotype, or biochemical and imaging findings [16]. Notably, some patients have shown normal brain imaging and plasma levels of ornithine and ammonia despite severe clinical symptoms [17, 18]. The complex pathogenesis of HHH syndrome contributes to the challenges in managing metabolic disorders in affected patients.

The optimal treatment for metabolic disorders involves addressing the underlying metabolic defects. LT has emerged as a potentially curative intervention for various UCDs, particularly in preventing endogenous catabolism and hyperammonemia [19]. However, a key distinction in HHH syndrome is that metabolic deficiencies, such as ornithine transport disorders, may persist outside the liver following transplantation [16]. Although ORC1 deficiency is systemic, the liver plays a dominant role in ammonia detoxification and ornithine metabolism. By restoring functional ornithine transport in hepatocytes, LT may reduce hyperammonemic episodes and stabilize metabolic homeostasis, even if extrahepatic deficiencies persist. This hepatic correction could mitigate secondary neurotoxicity from recurrent metabolic crises, which may explain the observed stabilization or improvement in neurological function despite ongoing extrahepatic defects. However, this mechanism remains speculative and warrants further investigation. Due to the rarity of HHH syndrome, only two patients have undergone liver transplantation for recurrent hyperammonemia and acute encephalopathy. De Bruyne et al. described a boy transplanted at 7 years for recurrent hyperammonemia; at 12-year follow-up, ammonia and amino acids remained normal on an unrestricted diet, with stable cognition but persistent spastic diplegia. This case demonstrated the metabolic benefit of LT but did not achieve reversal of established neurological deficits, and lacked detailed longitudinal neuroimaging or standardized functional assessments [8]. Wan et al. reported a neonatal case with acute liver failure secondary to HHH syndrome who underwent LT; however, the absence of long-term follow-up data on metabolic parameters and neurological outcomes precludes meaningful conclusions about efficacy [9]. In contrast, our study represents the largest single-center series to date, with comprehensive perioperative biochemical monitoring and longitudinal neurological assessments. Our findings confirm that LT can achieve durable metabolic control without dietary restriction or ammonia scavengers, and suggest that early LT—before irreversible neurological injury—may improve functional outcomes. These results not only address the evidence gap left by previous isolated case reports but also provide clinically relevant guidance for timing and indications of LT in HHH syndrome. Similar outcomes have been reported in other metabolic disorders such as familial hypercholesterolemia [20] and methylmalonic acidemia [21–23], highlighting the effectiveness of liver transplantation as a treatment. However, this remains speculative, as our study did not directly assess metabolic function in extrahepatic tissues.

Whether LT can improve neurological symptoms in patients with UCDs remains controversial [24, 25]. Our case studies indicate that LT can effectively halt the progression of spastic paraplegia compared to conservative management. While patients with longstanding spastic paraplegia may not experience significant gait improvements, those in the early stages of this condition can achieve marked recovery. For example, case 6 who developed an aversion to protein-rich foods at six months and was diagnosed with HHH syndrome at 26 months received conservative treatment but progressed to severe spastic paraplegia. After undergoing LDLT just 4 days after the onset of symptoms, the patient’s condition improved significantly, allowing him to walk independently within two weeks. In approximately three months, his motor function had essentially returned to normal, with no impact on his intellectual development.

The mechanism underlying spastic paraplegia in HHH syndrome remains unclear and does not appear solely linked to hyperammonemia, as similar manifestations are not observed in other UCDs except for argininaemia. Some studies suggest that the accumulation of polyamines may contribute to neurological damage through alternative metabolic pathways [26]. We hypothesize that traditional treatments focusing on hyperammonemia may overlook the impact of other metabolites involved in the urea cycle. Given that neurons are permanent cells, prolonged spastic paraplegia may lead to irreversible neuronal damage, making recovery unlikely. Thus, early liver transplantation could prevent further neuronal death and lead to better outcomes. We advocate for LT before irreversible neurological damage occurs, particularly for patients showing poor responses to conservative management or experiencing recurrent metabolic crises. In selected cases, presentation of poor treatment compliance, diet-induced growth retardation, poor school attendance, altered psychological status or problems with familial and social integration, which predicts worse prognosis and quality of life, may also be indications [16]. Ideally, LT should be performed as soon as indication exists to achieve the best neurological outcome [27].

With the development of liver transplantation and antirejection therapy, surgical complications are no longer the major contradiction in treatment. Reported posttransplant survival in ornithine transcarbamylase deficiency and in non-UCD patients has been the same, attaining 95% at one year and 90% at 5 years in large pediatric liver transplantation programs [19, 28, 29], exceeding the overall survival rate of urea circulation disorder and HHH syndrome. The dosage of immunosuppressive agents for recipients with metabolic disease is reduced more rapidly and can usually be converted into tacrolimus monotherapy within the first year, while the concentration of FK506 can be maintained at 6–8 ng/ml and gradually decreased to less than 3 ng/ml within 5 years. Low drug concentrations reduce the risk of infection and other adverse reactions, allowing patients to achieve good quality of life and social adaptation. However, one point that needs to be noted is that the success rate of liver transplantation is higher in patients over 3 months of age and over 5 kg of body weight than in those who are do not meet these criteria [30]. Therefore, although no absolute contraindication exists in children at birth and during infancy, the timing of liver transplantation still requires a comparison of mortality from primary disease and surgical risk, as well as the experience and skills of the transplant centers.

Due to limitations in testing methods, we did not acquire data on urinary homocitrulline data. Moreover, the lack of systematic neuroimaging and neurocognitive follow-up restricts our ability to determine whether neurological improvements were attributable solely to hepatic metabolic correction or were influenced by ongoing extrahepatic pathology. Given the systemic nature of ORC1 deficiency, the assumption that LT alone can fully correct the metabolic defect remains speculative. Future studies incorporating standardized imaging protocols, neurodevelopmental assessments, and evaluation of extrahepatic metabolic function will be essential to clarify the neurological benefits of LT in this patient population. Given the small sample size, further studies are needed to clarify the relationships between preoperative metabolic factors, imaging results, and patient prognosis. Nevertheless, previous cases indicate that severe disability does not preclude successful outcomes. This study represents the largest single-center experience with HHH syndrome. Together with previous cases, our findings suggest that liver transplantation may serve as a curative treatment, enabling patients to return to normal diets, enhance psychomotor development and social integration, particularly for those facing growth retardation or metabolic crises due to strict dietary limitations.

## Supplementary Information

Below is the link to the electronic supplementary material.

Supplementary Material 1

## Data Availability

The datasets generated and analyzed during the current study are available from the corresponding author on reasonable request.
