# Supplementary material for: Liver transplantation can prevent the progression of neurological damage in hyperornithinemia-hyperammonemia-homocitrullinuria syndrome and maintain long-term metabolic stability — The largest single-center experience
Source: Orphanet J Rare Dis. 2025 Oct 22;20:532. doi: 10.1186/s13023-025-04077-5 (PMC12542414; doi:10.1186/s13023-025-04077-5)
Supplement: Supplementary file 1 — Supplementary Material 1 [file 13023_2025_4077_MOESM1_ESM.docx]

| Case 1 | | | |
| --- | --- | --- | --- |
| Category | Score 5 | Score 3 | Score 1 |
| **Original disease** |  |  |  |
| Liver-oriented disease | ✓ |  |  |
| Previous case report |  | ✓ |  |
| **Effectiveness of medical treatment** |  |  |  |
| Metabolic decompensation which necessitated hospitalization ≥6 times/yr | ○ |  |  |
| Metabolic decompensation which necessitated hospitalization 3–5 times/yr |  | ✓ |  |
| Metabolic decompensation which necessitated admission ≥6 times/yr |  |  | ○ |
| Metabolic decompensation which necessitated ICU care with apheresis ≥2 times/yr | ○ |  |  |
| Extremely poor response/adherence for medical treatment |  | ○ |  |
| Poor response/adherence for medical treatment |  |  | ✓ |
| **Quality of life** |  |  |  |
| Nasogastric tube feeding/frequent meal |  | ○ |  |
| Progressive neurological impairment |  | ✓ |  |
| **Present status** |  |  |  |
| Good social interaction, full ambulation, partially impaired gross and fine motor skills, use of language, mildly delayed development, only modest learning deficits |  |  | ○ |
| Growth retardation (height<2.5 s.d.) |  |  | ○ |
| Continuous abnormal laboratory test (NH3, lactate, base excess, liver function, cholesterol, glucose) |  | ✓ |  |

Score interpretation: 18

≥10: Absolute indication for liver transplantation

10 > score ≥ 5: Relative indication

5 > score ≥ 3: Prudence indication

< 3: Contraindication

| Case 2 | | | |
| --- | --- | --- | --- |
| Category | Score 5 | Score 3 | Score 1 |
| **Original disease** |  |  |  |
| Liver-oriented disease | ✓ |  |  |
| Previous case report |  | ✓ |  |
| **Effectiveness of medical treatment** |  |  |  |
| Metabolic decompensation which necessitated hospitalization ≥6 times/yr | ○ |  |  |
| Metabolic decompensation which necessitated hospitalization 3–5 times/yr |  | ✓ |  |
| Metabolic decompensation which necessitated admission ≥6 times/yr |  |  | ○ |
| Metabolic decompensation which necessitated ICU care with apheresis ≥2 times/yr | ✓ |  |  |
| Extremely poor response/adherence for medical treatment |  | ✓ |  |
| Poor response/adherence for medical treatment |  |  | ○ |
| **Quality of life** |  |  |  |
| Nasogastric tube feeding/frequent meal |  | ○ |  |
| Progressive neurological impairment |  | ✓ |  |
| **Present status** |  |  |  |
| Good social interaction, full ambulation, partially impaired gross and fine motor skills, use of language, mildly delayed development, only modest learning deficits |  |  | ○ |
| Growth retardation (height<2.5 s.d.) |  |  | ○ |
| Continuous abnormal laboratory test (NH3, lactate, base excess, liver function, cholesterol, glucose) |  | ✓ |  |

Score interpretation: 25

≥10: Absolute indication for liver transplantation

10 > score ≥ 5: Relative indication

5 > score ≥ 3: Prudence indication

< 3: Contraindication

| Case 3 | | | |
| --- | --- | --- | --- |
| Category | Score 5 | Score 3 | Score 1 |
| **Original disease** |  |  |  |
| Liver-oriented disease | ✓ |  |  |
| Previous case report |  | ✓ |  |
| **Effectiveness of medical treatment** |  |  |  |
| Metabolic decompensation which necessitated hospitalization ≥6 times/yr | ○ |  |  |
| Metabolic decompensation which necessitated hospitalization 3–5 times/yr |  | ✓ |  |
| Metabolic decompensation which necessitated admission ≥6 times/yr |  |  | ○ |
| Metabolic decompensation which necessitated ICU care with apheresis ≥2 times/yr | ○ |  |  |
| Extremely poor response/adherence for medical treatment |  | ○ |  |
| Poor response/adherence for medical treatment |  |  | ✓ |
| **Quality of life** |  |  |  |
| Nasogastric tube feeding/frequent meal |  | ○ |  |
| Progressive neurological impairment |  | ✓ |  |
| **Present status** |  |  |  |
| Good social interaction, full ambulation, partially impaired gross and fine motor skills, use of language, mildly delayed development, only modest learning deficits |  |  | ○ |
| Growth retardation (height<2.5 s.d.) |  |  | ○ |
| Continuous abnormal laboratory test (NH3, lactate, base excess, liver function, cholesterol, glucose) |  | ○ |  |

Score interpretation: 15

≥10: Absolute indication for liver transplantation

10 > score ≥ 5: Relative indication

5 > score ≥ 3: Prudence indication

< 3: Contraindication

| Case 4 | | | |
| --- | --- | --- | --- |
| Category | Score 5 | Score 3 | Score 1 |
| **Original disease** |  |  |  |
| Liver-oriented disease | ✓ |  |  |
| Previous case report |  | ✓ |  |
| **Effectiveness of medical treatment** |  |  |  |
| Metabolic decompensation which necessitated hospitalization ≥6 times/yr | ✓ |  |  |
| Metabolic decompensation which necessitated hospitalization 3–5 times/yr |  | ○ |  |
| Metabolic decompensation which necessitated admission ≥6 times/yr |  |  | ○ |
| Metabolic decompensation which necessitated ICU care with apheresis ≥2 times/yr | ○ |  |  |
| Extremely poor response/adherence for medical treatment |  | ○ |  |
| Poor response/adherence for medical treatment |  |  | ✓ |
| **Quality of life** |  |  |  |
| Nasogastric tube feeding/frequent meal |  | ○ |  |
| Progressive neurological impairment |  | ✓ |  |
| **Present status** |  |  |  |
| Good social interaction, full ambulation, partially impaired gross and fine motor skills, use of language, mildly delayed development, only modest learning deficits |  |  | ○ |
| Growth retardation (height<2.5 s.d.) |  |  | ○ |
| Continuous abnormal laboratory test (NH3, lactate, base excess, liver function, cholesterol, glucose) |  | ○ |  |

Score interpretation: 17

≥10: Absolute indication for liver transplantation

10 > score ≥ 5: Relative indication

5 > score ≥ 3: Prudence indication

< 3: Contraindication

| Case 5 | | | |
| --- | --- | --- | --- |
| Category | Score 5 | Score 3 | Score 1 |
| **Original disease** |  |  |  |
| Liver-oriented disease | ✓ |  |  |
| Previous case report |  | ✓ |  |
| **Effectiveness of medical treatment** |  |  |  |
| Metabolic decompensation which necessitated hospitalization ≥6 times/yr | ○ |  |  |
| Metabolic decompensation which necessitated hospitalization 3–5 times/yr |  | ✓ |  |
| Metabolic decompensation which necessitated admission ≥6 times/yr |  |  | ○ |
| Metabolic decompensation which necessitated ICU care with apheresis ≥2 times/yr | ○ |  |  |
| Extremely poor response/adherence for medical treatment |  | ○ |  |
| Poor response/adherence for medical treatment |  |  | ✓ |
| **Quality of life** |  |  |  |
| Nasogastric tube feeding/frequent meal |  | ○ |  |
| Progressive neurological impairment |  | ✓ |  |
| **Present status** |  |  |  |
| Good social interaction, full ambulation, partially impaired gross and fine motor skills, use of language, mildly delayed development, only modest learning deficits |  |  | ○ |
| Growth retardation (height<2.5 s.d.) |  |  | ○ |
| Continuous abnormal laboratory test (NH3, lactate, base excess, liver function, cholesterol, glucose) |  | ○ |  |

Score interpretation: 15

≥10: Absolute indication for liver transplantation

10 > score ≥ 5: Relative indication

5 > score ≥ 3: Prudence indication

< 3: Contraindication

| Case 4 | | | |
| --- | --- | --- | --- |
| Category | Score 5 | Score 3 | Score 1 |
| **Original disease** |  |  |  |
| Liver-oriented disease | ✓ |  |  |
| Previous case report |  | ✓ |  |
| **Effectiveness of medical treatment** |  |  |  |
| Metabolic decompensation which necessitated hospitalization ≥6 times/yr | ✓ |  |  |
| Metabolic decompensation which necessitated hospitalization 3–5 times/yr |  | ○ |  |
| Metabolic decompensation which necessitated admission ≥6 times/yr |  |  | ○ |
| Metabolic decompensation which necessitated ICU care with apheresis ≥2 times/yr | ○ |  |  |
| Extremely poor response/adherence for medical treatment |  | ○ |  |
| Poor response/adherence for medical treatment |  |  | ✓ |
| **Quality of life** |  |  |  |
| Nasogastric tube feeding/frequent meal |  | ○ |  |
| Progressive neurological impairment |  | ✓ |  |
| **Present status** |  |  |  |
| Good social interaction, full ambulation, partially impaired gross and fine motor skills, use of language, mildly delayed development, only modest learning deficits |  |  | ○ |
| Growth retardation (height<2.5 s.d.) |  |  | ○ |
| Continuous abnormal laboratory test (NH3, lactate, base excess, liver function, cholesterol, glucose) |  | ○ |  |

Score interpretation: 17

≥10: Absolute indication for liver transplantation

10 > score ≥ 5: Relative indication

5 > score ≥ 3: Prudence indication

< 3: Contraindication
